# Unraveling the interplay: early-stage atrial functional mitral regurgitation and left atrial electrical substrate in atrial fibrillation patients

**DOI:** 10.3389/fcvm.2024.1382570

**Published:** 2024-08-22

**Authors:** Yazan Mohsen, Dennis Rottländer, Nora Großmann, Nicole Lewandowski, Marc Horlitz, Florian Stöckigt

**Affiliations:** ^1^ Department of Cardiology, Krankenhaus Porz am Rhein, Cologne, Germany; ^2^ Department of Cardiology, Faculty of Health, School of Medicine, University Witten/Herdecke, Witten, Germany; ^3^ Department of Internal Medicine II, University Hospital Bonn, Bonn, Germany

**Keywords:** atrial fibrillation, low voltage areas, atrial substrate, aFMR, atrial remodeling, atrial cardiomyopathy, mapping

## Abstract

**Background:**

Atrial fibrillation (AF) triggers atrial remodeling, impacting atrial function and ablation efficacy. This remodeling leads to atrial cardiomyopathy and dilatation, linked to mitral regurgitation, forming atrial functional mitral regurgitation (aFMR). Our study explores the relationship between early-stage-aFMR and the atrial electrical architecture, focusing on left atrial bipolar voltage and low-voltage areas (LVAs) in AF patients.

**Methods:**

We enrolled 282 patients undergoing redo-PVI after AF recurrence post-PVI. Echocardiography was performed prior to ablation, and only patients with no, mild, or mild-to-moderate aFMR were included. Ablation used radiofrequency and a 3D mapping system, with atrial voltage documented on each atrial wall. LVAs were calculated using high-density maps, and patients were followed for 15 months.

**Results:**

Significant differences in left atrial voltage and LVA extent were observed based on aFMR severity. Patients with aFMR 1 + had significantly lower atrial voltage compared to no-aFMR, but no significant increase in LVAs. Patients with aFMR 2 + showed lower voltage amplitudes in all atrial regions and larger LVAs compared to no-aFMR patients. AF recurrence was significantly higher in the aFMR group (62.9% vs. 48.3%, *p* = 0.027) within 1 year. aFMR was associated with AF recurrence after adjusting for sex, age, and AF types (HR: 1.517, 95% CI: 1.057–2.184, *p* = 0.025).

**Conclusion:**

aFMR in AF patients may indicate progressive atrial remodeling and left atrial cardiomyopathy, characterized by reduced atrial voltage and increased LVAs. aFMR is linked to PVI outcomes, suggesting its consideration in AF therapy decision-making.

## Introduction

Atrial fibrillation (AF) is one of the predominant arrhythmias of clinical relevance, with its incidence rising in parallel to the ageing of the global population (1). Intrinsically linked to AF is the phenomenon of atrial remodeling, which precipitates atrial cardiomyopathy and atrial dilatation, even in the absence of concomitant left ventricular (LV) dysfunction (2, 3).

Numerous studies have reported the presence of mitral regurgitation (MR) in cohorts with isolated AF (4–6). In these patients sinus rhythm restoration showed improvements of the MR (7). This observation has led to the characterization of atrial functional mitral regurgitation (aFMR). Characteristics of aFMR are normal LV-function, normal mitral leaflet motion, a more central regurgitation jet and severe left atrial dilatation (8).

In paroxysmal AF aFMR is associated with recurrent AF following catheter ablation, especially those with significant MR (9). Furthermore, once left atrial dilatation accompanied by significant aFMR occurred, neither mitral valve repair nor atrial fibrillation ablation strategies might be convincing therapeutic options (8). Hence early detection and treatment of aFMR are essential for the success of the treatment.

As the left atrium (LA) undergoes structural and electrical remodeling in the presence of AF, electroanatomical mapping has emerged as a pivotal tool in elucidating the extent of atrial myocardial damage represented by low voltage areas (LVAs) (10). These LVA not only are associated with compromised atrial functionality (11). They also have prognostic value, predicting the efficacy of catheter ablation interventions and long-term rhythm stability in AF cohorts (12). Recent data underscore the potential association between lower atrial voltages and the severity of mitral regurgitation (13).

In this study we aim to delve deeper into the relationship between early stages of aFMR (aFMR1 + and aFMR2+) and LA bipolar voltage along with the extent and localization of LVAs in AF patients.

## Methods

### Study design

This study is a retrospective analysis of 282 patients with recurrent paroxysmal and persistent AF, who underwent redo radiofrequency (RF) catheter ablation at a high-volume EP center (>1,000 ablations per year) in Germany. Patient characteristics, including age and comorbidities, were recorded. All patients underwent a previous PVI without substrate modification, with the median time between the initial procedure and the redo ablation being 2.1 years [interquartile range (IQR) 1.1–3.7 years]. During the redo ablation procedure, at least one reconnected PV was identified in all patients. Each patient subsequently received a re-PVI, where RF energy was applied exclusively at the reconnection gaps of the previous PVI line.

Follow-up visits were conducted after three months (blanking period) and 12 months following the conclusion of the blanking period. The study protocol was approved by the local ethical committee in adherence to the Declaration of Helsinki.

Only patients with no, mild (aFMR1+) or mild-to-moderate (aFMR2+) aFMR were included in this study. Patients were classified according to their aFMR status:

(i) No aFMR and trace aFMR = no-aFMR
(ii) Mild and mild-to-moderate aFMR (aFMR 1 + and 2+) = aFMR.

### Electroanatomic mapping

All procedures were conducted in deep sedation, with continuous monitoring of hemodynamic parameters. After positioning two diagnostic catheters in the CS (Dynamic XTTM, Boston Scientific; 6 F; 8 electrodes in 2-5-2 mm spacing) and in the right ventricular apex (SupremeTM, St. Jude Medical; 5 F; 4 electrodes at 5 mm intervals), two transseptal punctures were performed. Subsequently, the ablation catheter (THERMOCOOL SMARTTOUCH® Bi-Directional Catheter, Biosense Webster; 7.5 F) and a mapping catheter, either PENTARAY (20 Electrodes, 2–6–2 mm) or LASSO (20 Electrodes, Biosense Webster; 7 F), were introduced into the LA. The activated clotting time was maintained above 300 s.

Electroanatomic mapping was executed using the CARTO® 3 system during sinus rhythm. In instances where AF was observed at the onset of the procedure, an electrical cardioversion was performed to restore sinus rhythm. All analyzed maps consisted of a minimum of 1,000 electroanatomic points to ensure an even and dense sampling of the anterior, superior, posterior, septal, and left lateral walls of the atrium.

Bipolar voltage amplitude was recorded from various points on each atrial wall in all 284 maps. Subsequently, the mean signal amplitude was computed for each wall and the entire atria.

Out of the 284 maps, 84 HD maps generated using the Pentaray catheter were selected for an in-depth analysis to evaluate the area of LVA. For these maps, the minimum number of electroanatomical points was set at 1,500. Seamless mapping of the atrium was conducted with the fill and color threshold set to five, ensuring a minimum density of at least one point per 0.7 cm^2^. To reduce artifacts, the CARTO confidence mapping filters (cycle length & tissue proximity index) were utilized. Only low voltage regions ≥1 cm^2^ were identified as LVAs.

The point filter was adjusted to five, accepting only points within a 5 mm distance from the surface for map contribution. Respiration gating was executed to enhance the accuracy of the captured atrial geometry, which was obtained using the high adjustment settings of 17.

**Figure 1:**
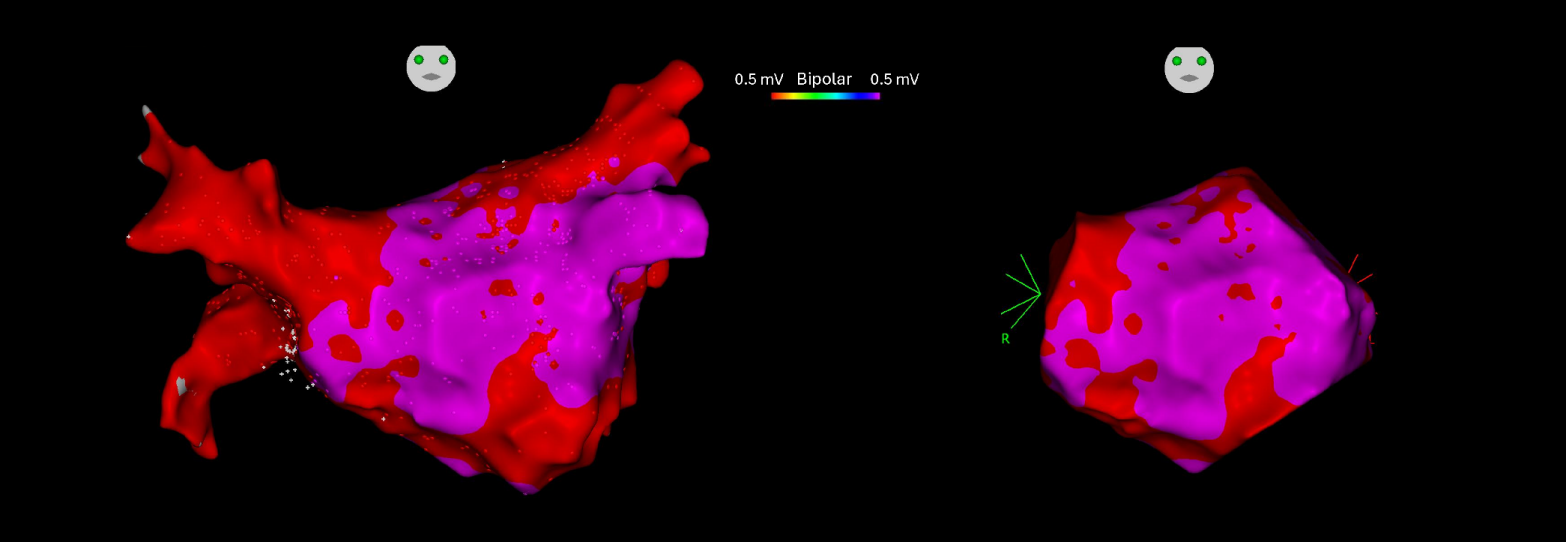
Electroanatomical mapping (EAM) 3D models of the left atrium before (left) and after (right) excluding the areas of pulmonary veins, mitral valve, and left atrial appendage generating the true left atrial surface (14).

The actual surface area of the LA was determined as previously described by excluding features such as the pulmonary veins, mitral valve, and left atrial appendage, and by disregarding areas associated with the remaining surfaces (refer to Figure 1) (14). This method facilitated an accurate calculation of the percentage of left atrial LVAs in relation to the true left atrial surface using the CARTO surface tool. LVAs near the PVs that might have resulted from prior ablation were excluded from the analysis.

### Echocardiography

Baseline two-dimensional echocardiography with color flow mapping was performed in every patient using a GE Vivid 9 echocardiography system (General Electrics, Chicago, USA). Two blinded echocardiographic experts reviewed all echocardiographic images and independently evaluated the severity of mitral regurgitation. Transesophageal echocardiography prior catheter ablation was performed to exclude left atrial thrombusand to ascertain functional mitral regurgitation. Patients without sinus rhythm, with degenerative mitral regurgitation, prior mitral valve surgery or FMR due to heart failure with reduced ejection fraction (HFrEF) were excluded. Vena contracta, MR jet area, LA area, MR/LA ratio were used for quantitative mitral valve assessment according to current recommendations (15).

For grading the severity of mitral regurgitation, a standard classification was used (16). The ratio of maximum MR color jet area to LA area (MR/LA ratio) was calculated and classification of MR was defined as mild MR (MR/LA ratio <0.2) and mild-to-moderate MR (MR/LA ratio ≥0.2 < 0.3). A vena contracta <3 mm and a jet area <4 cm^2^ was considered as aFMR1 + while a vena contracta ≥3 < 6 mm and a jet area ≥4 cm^2^ < 6 cm^2^ was classified as aFMR2 + . Determination of regurgitant volume using the proximal isovelocity surface area (PISA) method was only infrequently used in aFMR1 + due to small jets in mild MR. Therefore, it was not statistically evaluated in this study. Furthermore, anterior-posterior mitral annulus diameter was assessed and evaluated.

### Statistical methods

Data are presented as either mean ± standard deviation or, for non-normally distributed variables, as median with IQR. Two-group comparisons utilized the Mann-Whitney test, while the chi-squared tests catered to the analysis of categorical variables.

We probed associations between aFMR and AF recurrence employing Kaplan-Meier analysis, log-rank tests, and Cox proportional hazard regression across the entire patient cohort. Hazard ratios (HRs) were derived from three distinct Cox regression models:

1. Unadjusted.
2. Adjusted for age and sex.
3. Adjusted for age, sex, and AF type.

All statistical computations were performed using IBM SPSS Statistics Version 26 (IBM Inc., Armonk, NY, USA). A *P*-value of less than 0.05 was set as the threshold for statistical significance.

## Results

### Demographic and clinical characteristics related to aFMR

In our study cohort of 282 patients, there was a notable divergence in baseline characteristics based on aFMR status. Patients with aFMR (*n* = 122) were significantly older (median age 69 years) compared to those with no-aFMR (*n* = 160), who had a median age of 60.5 years (*p* < 0.001). The proportion of females was higher in the aFMR group (37.7%) than in the no-aFMR group (22.5%), with statistical significance (*p* = 0.005). Body mass index (BMI) and prevalence of diabetes mellitus also showed notable differences between the groups (See Table 1 for detailed baseline clinical characteristics).

**Table 1:** Baseline clinical characteristics.

	All (*n* = 282)	no-aFMR (*n* = 160)	aFMR (*n* = 122)	*P* ^a^
Age, years	64.0 [56.0–72.0]	60.5 [54.7–68.2]	69.0 [59.0–74.7]	0.000
Sex, female, *n* (%)	82 (29.1)	36 (22.5)	46 (37.7)	0.005
Body mass index, kg/m^2^	26.5 [24.4–29.7]	27.0 [25.0–30.0]	25.7 [24.0–29.0]	0.006
Diabetes mellitus, *n* (%)	25 (8.9)	20 (12.5)	5 (4.1)	0.045
Hypertension, *n* (%)	175 (62.1)	96 (60.0)	79 (64.8)	0.415
Persistent AF, *n* (%)	160 (56.7)	81 (50.6)	79 (64.8)	0.018
CHA2DS2VASc-score	2.6 [1.9–3.6]	2.0 [1.0–3.0]	2.5 [1.0–4.0]	0.000
OSAS, *n* (%)	33 (11.7)	17 (10.6)	16 (13.1)	0.519
Stroke/TIA, *n* (%)	31 (11.0)	14 (8.8)	17 (13.9)	0.168
EF%	55.0 [55.0–60.0]	55.0 [55.0–60.0]	55.0 [54.7–60.0]	0.409
Coronary heart disease, (%)	56 (19.9)	24 (15.0)	32 (26.2)	0.088
Total LVA area %	5.4 [1.7–14.8]	3.5 [1.2–10.2]	7.8 [2.1–21.0]	0.029
LA area cm^2^	18.4 [15.3–22.3]	17.0 [14.6–19.7]	20.6 [16.6–24.7]	0.000

OSA, obstructive sleep apnoea syndrome; EF, ejection fraction; TIA, transient ischemic attack; LVA, low voltage areas; LA, left atrium.

^a^ Comparison between no-aFMR and FMR groups.

A total of 253 patients were subject to follow-up, during which AF recurrence transpired in 138 individuals, constituting 54.5% of the entire cohort. The median follow-up duration was 14.5 [3.7–15.0] months.

### Echocardiographic analysis of aFMR

**Figure 2:**
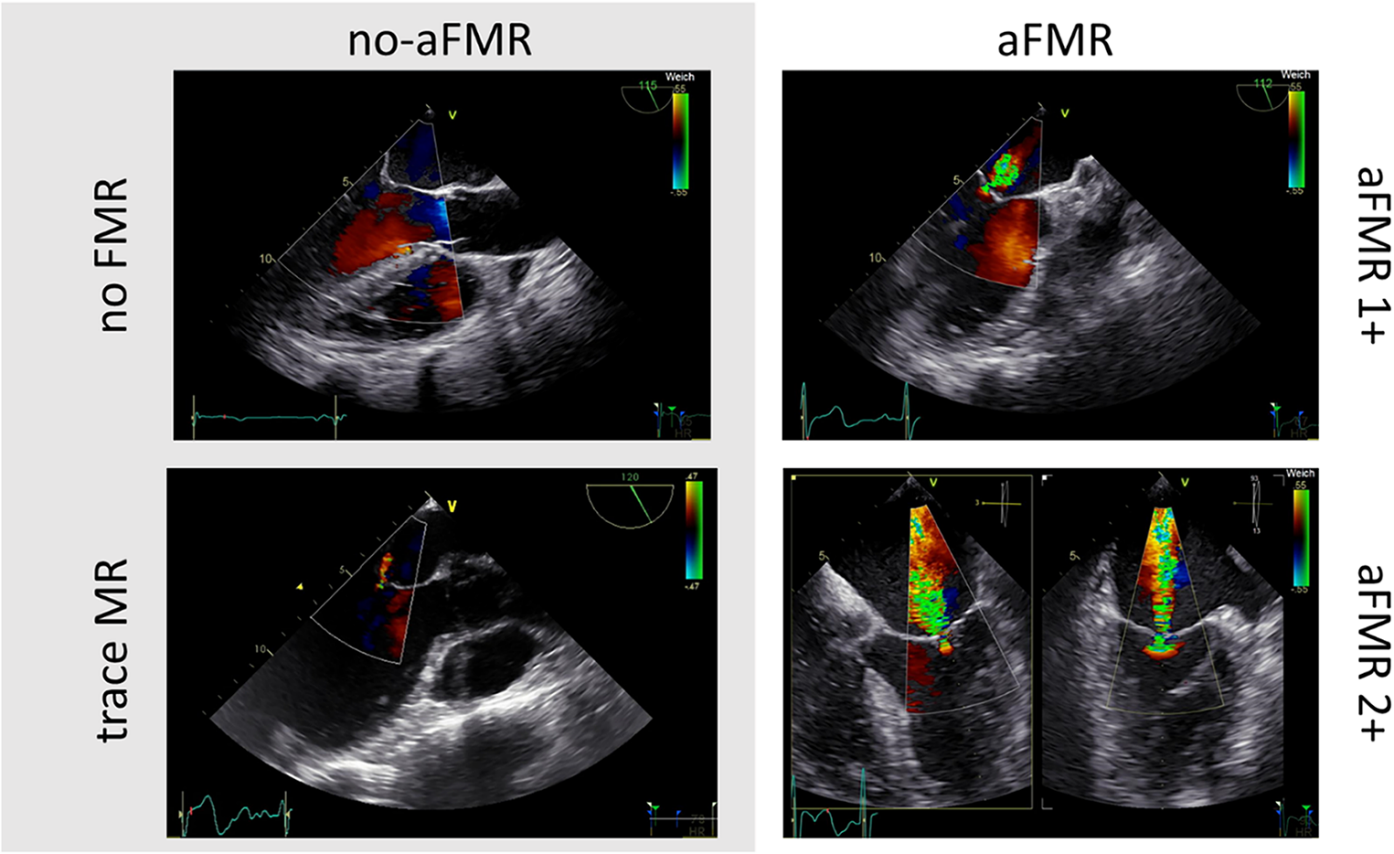
Transesophageal echocardiographic imaging of patients with no or trace aFMR (left) and the aFMR ≥1 + group (right).

**Figure 3:**
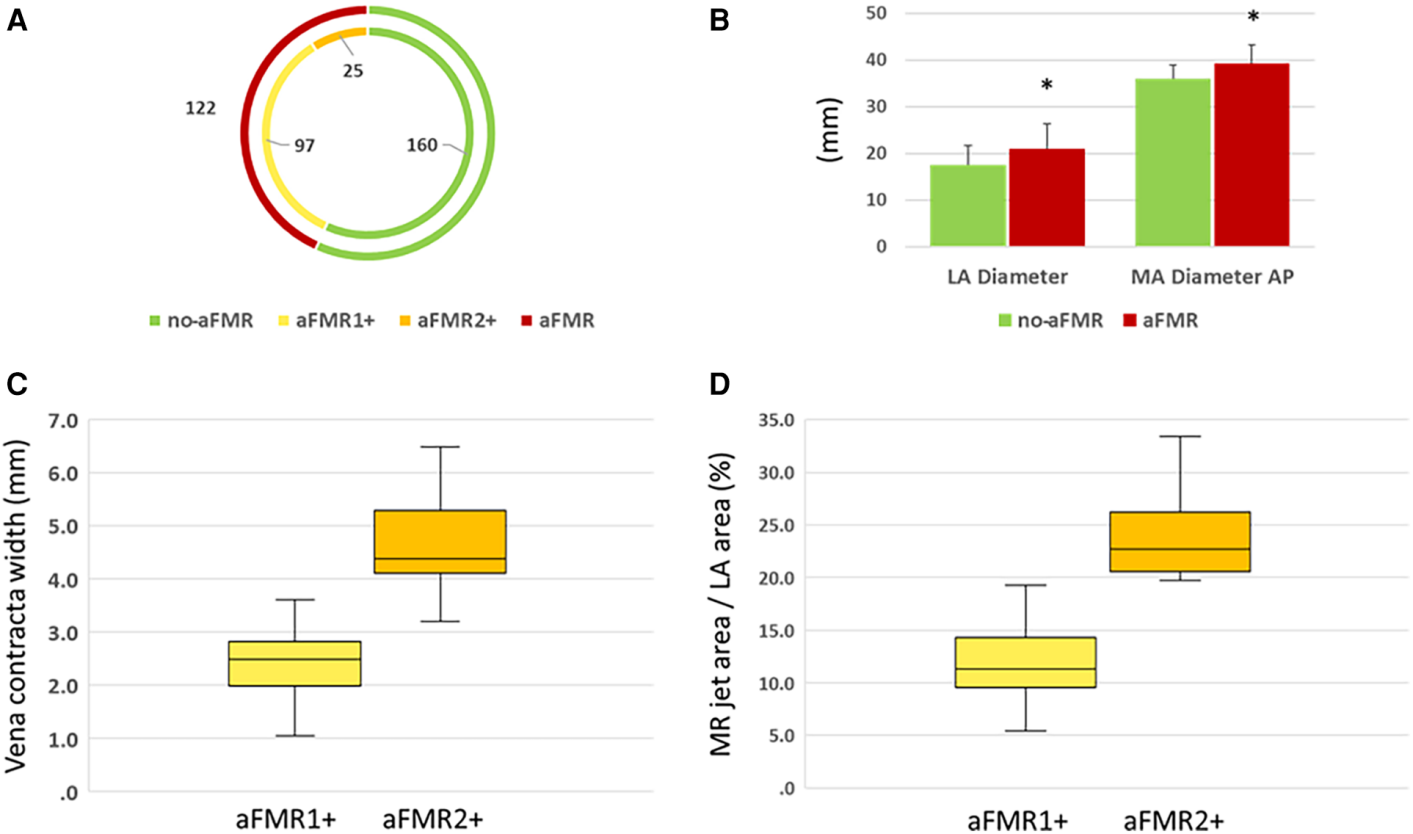
**(A)** The distribution of aFMR across the examined cohort; **(B)** left atrial (LA) and anterior to posterior (AP) Mitral Annulus (MA) Diameter variation; **(C)** vena contracta width & **(D)** mitral regurgitation jet area per left atrial area variation between no-aFMR and aFMR group.

Figure 2 shows original transesophageal echocardiographic imaging of patients with no or trace aFMR (no-aFMR group) and the aFMR group (aFMR1 + and aFMR2+). In 160 patients echocardiographic evaluation revealed no or trace aFMR (no-aFMR group), while aFMR1 + was found in 97 patients and aFMR 2 + in 25 patients (122 patients, aFMR group, Figure 3A). aFMR was associated with significantly higher LA diameter and anterior-posterior mitral annulus diameter (Figure 3B). As per definition in aFMR1 + vena contracta width and mitral regurgitation jet area per left atrial area were significantly smaller compared to aFMR2+ (Figures 3C,D).

### Association between left atrial voltage and aFMR

Analysis of left atrial voltage highlighted significant differences between the two cohorts. Patients in the aFMR group showed reduced average voltage amplitudes in the anterior, posterior, and roof regions compared to those in the no-aFMR group. Moreover, a notably higher percentage of patients in the aFMR group had at least two points with voltages below 0.5 mV in these areas (anterior, posterior, or roof), in contrast to their counterparts in the no-aFMR group. Detailed values and the statistical significance of these observations are presented in Table 2A.

**Table 2A:** Left atrial voltage and low voltage areas in the no-aFMR and aFMR groups.

	All (*n* = 282)	no-aFMR (*n* = 160)	aFMR (*n* = 122)	*P* ^a^
Anterior voltage (mV)		1.4 [0.8–2.2]	1.1 [0.5–1.7]	0.007
Posterior voltage (mV)		1.9 [1.0–3.1]	0.9 [0.7–2.0]	0.002
Roof voltage (mV)		1.2 [0.7–1.9]	0.9 [0.4–1.4]	0.000
Average all points (mV)		1.4 [0.9–2.2]	1.1 [0.6–1.9]	0.000
Anterior 2 points <0.5 mV (%)		24 (15.0%)	44 (36.6%)	0.000
Posterior 2 points <0.5 mV (%)		14 (8.7%)	27 (22.1%)	0.002
Roof 2 points <0.5 mV (%)		31 (19.3%)	49 (40.1%)	0.001

	All (*n* = 84)	no-aFMR (*n* = 46)	aFMR (*n* = 38)	
Anterior LVA area %		1.1 [0.0–3.9]	2.7 [0.2–3.9]	0.066
Posterior LVA area %		0.0 [0.0–1.6]	0.8 [0.0–4.3]	0.031
Roof LVA area %		1.2 [0.0–3.5]	2.6 [0.4–5.3]	0.036
Septum LVA area %		1.1 [0.0–3.6]	3.2 [0.0–5.4]	0.026
Total LVA area %		3.5 [1.2–10.2]	7.8 [2.1–21.0]	0.029

LVA, low voltage areas.

^a^ Comparison between no-aFMR and FMR groups.

Further analysis of the individual aFMR 1 + and aFMR 2 + groups revealed that this reduction in voltage was consistent in both groups when compared to the no-aFMR group, as detailed in Tables 2B.

**Table 2B:** Left atrial voltage and low voltage areas in in the no-aFMR and aFMR 1 + and aFMR 2 + groups.

	no-aFMR (*n* = 160)	aFMR 1 + (*n* = 97)	*P* ^b^	aFMR 2 + (*n* = 25)	*P* ^a^
Anterior voltage (mV)	1.4 [0.8–2.2]	1.1 [0.5–1.8]	0.059	0.6 [0.4–1.4]	0.003
Posterior voltage (mV)	1.9 [1.0–3.1]	1.5 [0.9–2.5]	0.020	1.31 [0.5–2.4]	0.004
Roof voltage (mV)	1.2 [0.7–1.9]	0.9 [0.54–1.5]	0.002	0.7 [0.4–1.2]	0.006
Average all points (mV)	1.4 [0.9–2.2]	1.2 [0.8–2.0]	0.035	0.8 [0.4–1.4]	0.001
Anterior 2 points <0.5 mV (%)	24 (15.0%)	29 (29.8%)	0.002	15 (60.0%)	0.000
Posterior 2 points <0.5 mV (%)	14 (8.7%)	18 (18.5%)	0.022	9 (36.0%)	0.000
Roof 2 points <0.5 mV (%)	31 (19.3%)	32 (32.9%)	0.041	17 (68.0%)	0.000

	no-aFMR (*n* = 46)	aFMR 1+ (*n* = 27)		aFMR 2+ (*n* = 11)	
Anterior LVA area %	1.1 [0.0–3.9]	2.7 [0.2–2.6]	0.160	8.3 [0.0–15.3]	0.098
Posterior LVA area %	0.0 [0.0–1.6]	0.6 [0.0–1.7]	0.145	4.1 [0.0–9.6]	0.018
Roof LVA area %	1.2 [0.0–3.5]	1.8 [0.0–3.9]	0.391	4. 5 [0.5–5.4]	0.025
Septum LVA area %	1.1 [0.0–3.6]	2.1 [0.0–5.2]	0.143	5.1 [2.1–9.2]	0.015
Total LVA area %	3.5 [1.2–10.2]	5.5 [1.9–17.8]	0.140	21.9 [3.1–38.4]	0.022

^a^ Comparison between no-aFMR and aFMR 2 + groups.

^b^ Comparison between no-aFMR and aFMR 1 + groups.

### LVA changes in relation to aFMR

**Figure 4:**
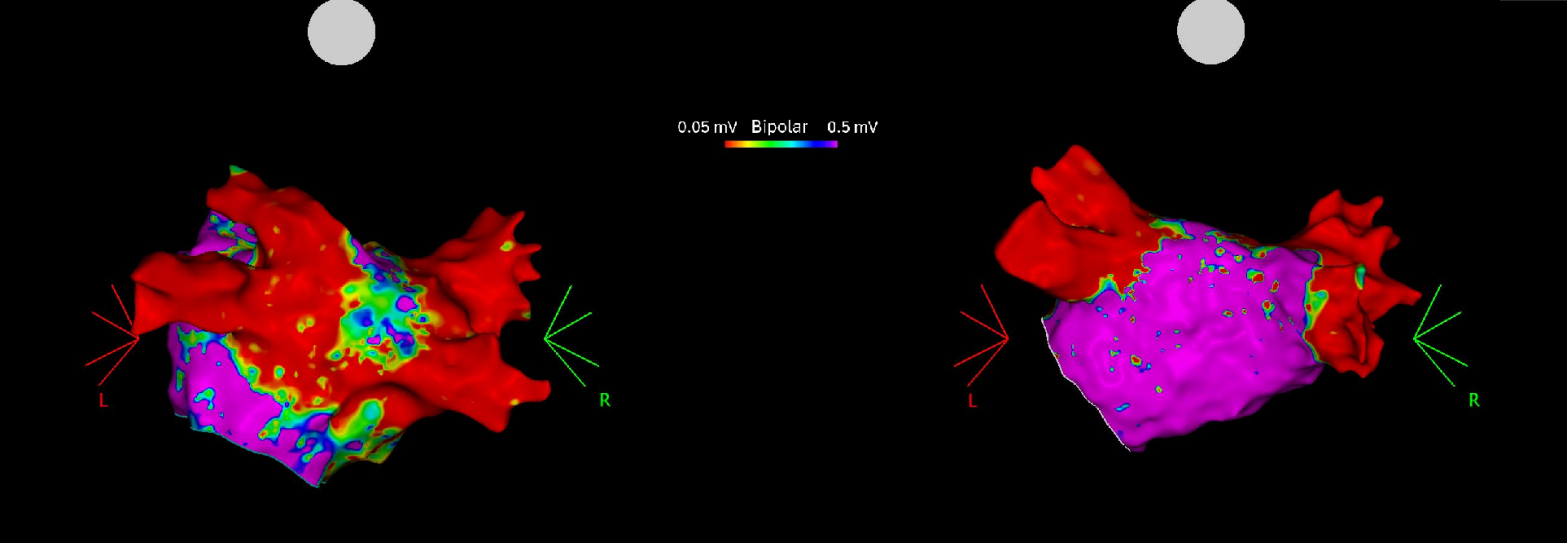
High density electroanatomical maps of LA showing Low voltage areas (LVA) on the posterior wall in an aFMR 2 + patient (left) and no LVAs in a no-aFMR patient (right).

In terms of LVA, patients with aFMR demonstrated significantly larger LVAs in the posterior wall, atrial roof, and septum compared to the no-aFMR group. Detailed data on these findings can be found in Table 2A. Original CARTO images of the differences in LVA observed between the two groups are presented in Figure 4.

Upon further comparison, no significant differences in LVAs were observed between the no-aFMR group and the aFMR 1 + group, as indicated in Table 2B. Conversely, a comparison between the no-aFMR and aFMR 2 + groups revealed a significant increase in LVA in the posterior wall, atrial roof, and septum, despite the limited sample size. These results are detailed in Table 2B.

### Impact of aFMR on atrial fibrillation recurrence

**Figure 5:**
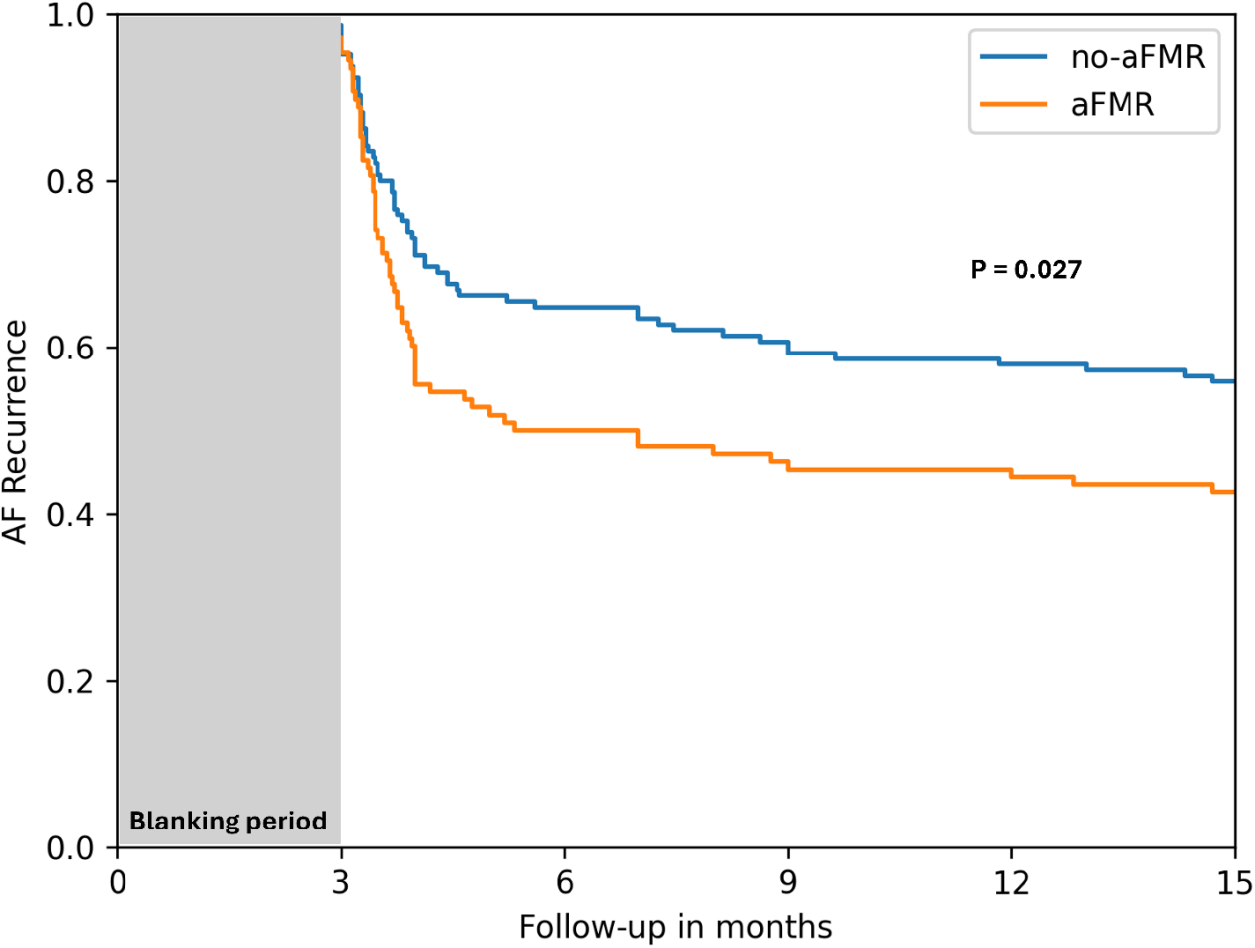
Kaplan-Meier survival curves depicting the time to AF recurrence for no-aFMR and aFMR groups.

The recurrence of AF was compared between the two groups using Kaplan-Meier analysis and log-rank tests (Figure 5). The aFMR group showed significantly higher rate of AF recurrence with of 62.9% after a follow-up time of 1 year after the blanking period vs. 48.3% in no-aFMR (*p* = 0.027).

The Cox regression analysis revealed a significant association between AF recurrence and aFMR, which persisted after adjustments for variables such as sex, age, and AF type. The hazard ratio for AF recurrence in the fully adjusted model (Model 3) was 1.517 with a 95% confidence interval of 1.057–2.184 (*P* = 0.025), as detailed in Table 3.

**Table 3:** Unadjusted and adjusted HRs for AF recurrence in the aFMR group.

All (*n* = 253)	HR^a^	95% CI		*P*
Model 1	1.587	1.116	2.256	0.010
Model 2	1.639	1.141	2.355	0.008
Model 3	1.517	1.057	2.184	0.025

Model 1 is unadjusted.

Model 2 adjusts to age and sex.

Model 3 adjusts for covariates in model 2 and AF type.

CI, confidence interval.

^a^ Hazard ratio (HR) is calculated with aFMR as the reference group.

## Discussion

Our findings reveal that patients with early-stage aFMR (1 + and 2+) displayed a discernibly reduced left atrial voltage. Significantly, this trend was not restricted to patients with persistent AF but was also evident in those diagnosed with paroxysmal AF. Additionally, a heightened prevalence of LVAs was observed within the aFMR group.

Several studies have documented that aFMR and LVA are more prevalent in women, older individuals, patients with DM, and those with a large left atrium (17–22). Our results are mostly consistent with these findings, as the aFMR group, which had significantly higher LVA, was older with a higher incidence of persistent AF and a greater proportion of women. However, the mentioned studies have shown an association between aFMR and LVAs and DM, which was not evident in our study. This could be attributed to two reasons: firstly, recent findings highlighted that LVAs were found more frequently in patients with DM and poor glycemic control, with HbA1c ≥7% being an independent predictor of LVAs (23). Secondly, while aFMR has been associated with DM, both heart failure with preserved ejection fraction (HFpEF) and AF are underlying causes of aFMR (24). While HFpEF is strongly linked to diabetes (25), whereas diabetes is less common in AF patients undergoing PVI (7), consistent with the prevalence of DM in our cohort.

Our study also observed region-specific differences in LVA distribution. While the anterior wall showed no significant difference in LVA, substantial increases were noted in the posterior wall, septum, and atrial roof in patients with mild to moderate aFMR. This pattern aligns with previous research that reports a higher prevalence of LVAs in the posterior wall among AF patients (26). Such observations underscore the intricate nature of the structural changes accompanying aFMR, particularly highlighting a more pronounced impact on the posterior wall as compared to the anterior wall.

Our comparative analysis between the no-aFMR group and aFMR1 + patients showed noticeable decline in voltage yet without significant LVA disparities. In contrast, patients with aFMR2 + additionally demonstrated significantly larger LVAs. This suggests a correlation between the severity of MR and the extent of atrial remodeling. Furthermore, even the presence of mild aFMR in AF patients might be indicative of progressive left atrial cardiomyopathy characterized by a decrease in atrial voltage indicating a pre-stage for LVA.

In our study cohort, we only included patients who encountered AF recurrence following an initial PVI and exhibited reconnected pulmonary veins. This selection criteria could potentially account for the observed higher rate of AF recurrence following the redo ablation procedure. The reconnected veins were re-isolated during the subsequent ablation procedure. This context suggests that in these patients, AF recurrence is more likely attributable to alterations in the atrial substrate rather than pulmonary vein triggers. Consistently, our analysis revealed a significant association between aFMR and AF recurrence. This finding aligns with the well-established relationship between the atrial substrate, atrial dilatation, and the likelihood of AF recurrence.

The progression of aFMR is multifaceted, involving AF initiated structural changes such as LA and annular dilatation accompanied with insufficient leaflet remodeling which contributes to the severity of atrial functional MR (27) as the dilatation of the annulus was not accompanied with the leaflet enlargement normally observed in healthy individuals (28). Furthermore, the functional changes in LA contractility due to the interstitial fibrosis and the following decrease in LA voltage and resulting LVAs presents a possible contributing factor to the development of aFMR as several studies suggested that the loss of atrial systole was associated with insufficient mitral valve closure in diastole and early systole resulting in aFMR (29, 30). Furthermore, the concomitant exacerbation of LA dysfunction by aFMR suggests a feedback loop that potentiates the deterioration of both LA function and aFMR severity.

While the causal relationship remains ambiguous, some studies (7, 31), have highlighted improvements in aFMR following the restoration of sinus rhythm. This suggests potential therapeutic benefits in mitigating left atrial remodeling associated with AF, thereby potentially alleviating aFMR. This hypothesis gains further credence from recent findings, which demonstrate a significant decrease in LVA after sinus rhythm restoration (32). Notably, the reversibility of early-stage remodeling (33) implies that both the presence and stage of aFMR should be considered in AF treatment strategies.

This study, however, has limitations. As a retrospective, single-center investigation that enrolled only patients undergoing repeat PVI, its findings may not be generalizable to patients who are PVI naive. Furthermore, evaluating LVA in this cohort is susceptible to errors, as distinguishing LVAs resulting from the previous PVI from those due to remodeling of the LA can be challenging.

In conclusion, the presence of early-stage aFMR in AF patients may serve as a marker of progressive atrial remodeling and the onset of left atrial cardiomyopathy, characterized by reduced atrial voltage and increased LVAs. Additionally, aFMR is linked to the outcomes of PVI, suggesting that even early-stages of aFMR should be considered in the decision-making process for AF therapy. Further research is needed to explore the potential reversibility of these changes.

## Data Availability

The data are available upon reasonable request to the authors and with the appropriate approval from the relevant ethics committee.
